# Supplementary figures and images for: CRISPR-Cas9 Screening of Kaposi’s Sarcoma-Associated Herpesvirus-Transformed Cells Identifies XPO1 as a Vulnerable Target of Cancer Cells
Source: mBio. 2019 May 14;10(3):e00866-19. doi: 10.1128/mBio.00866-19 (PMC6520457; doi:10.1128/mBio.00866-19)

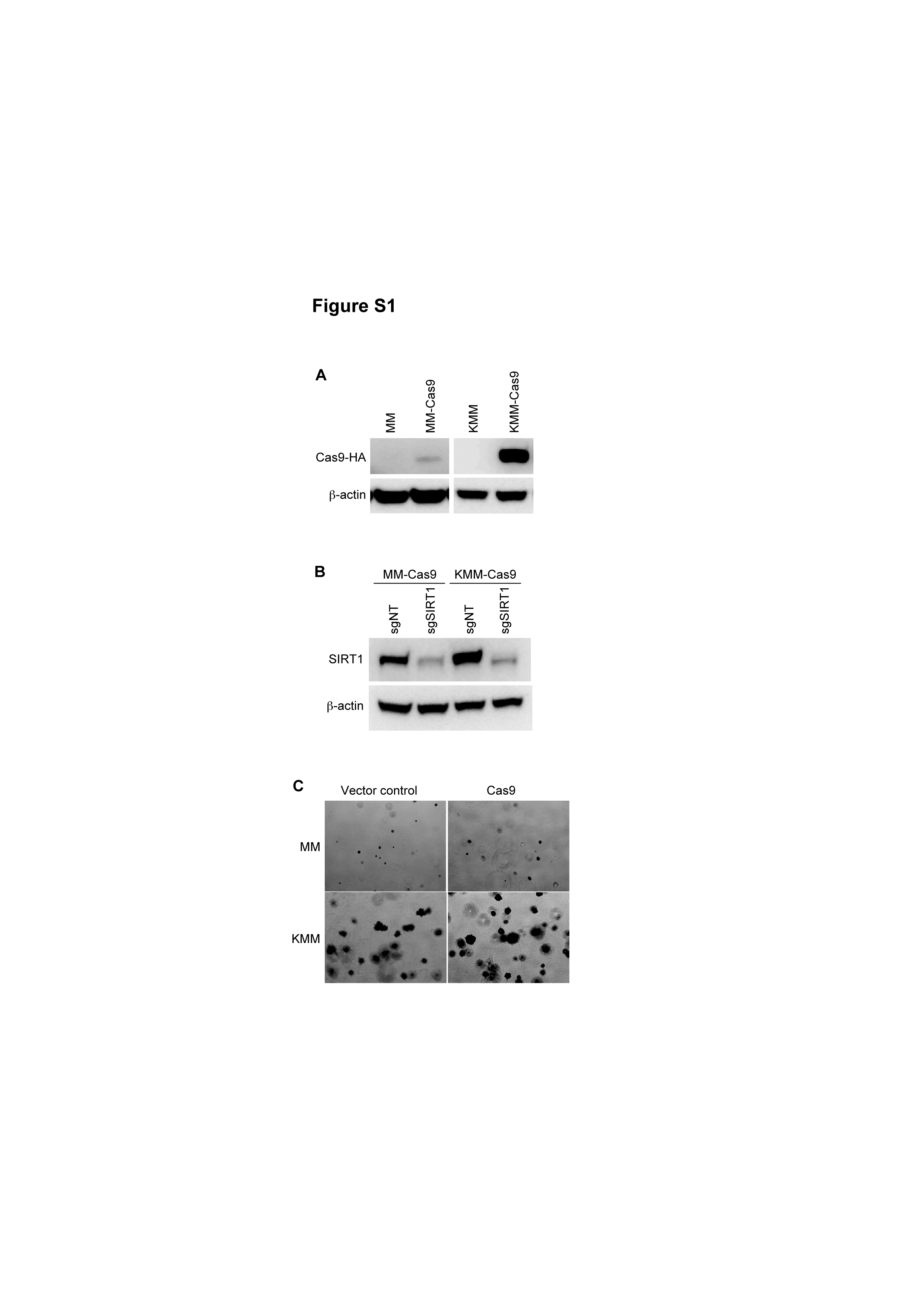

Supplement: FIG S1 [file mBio.00866-19-sf001.tif]

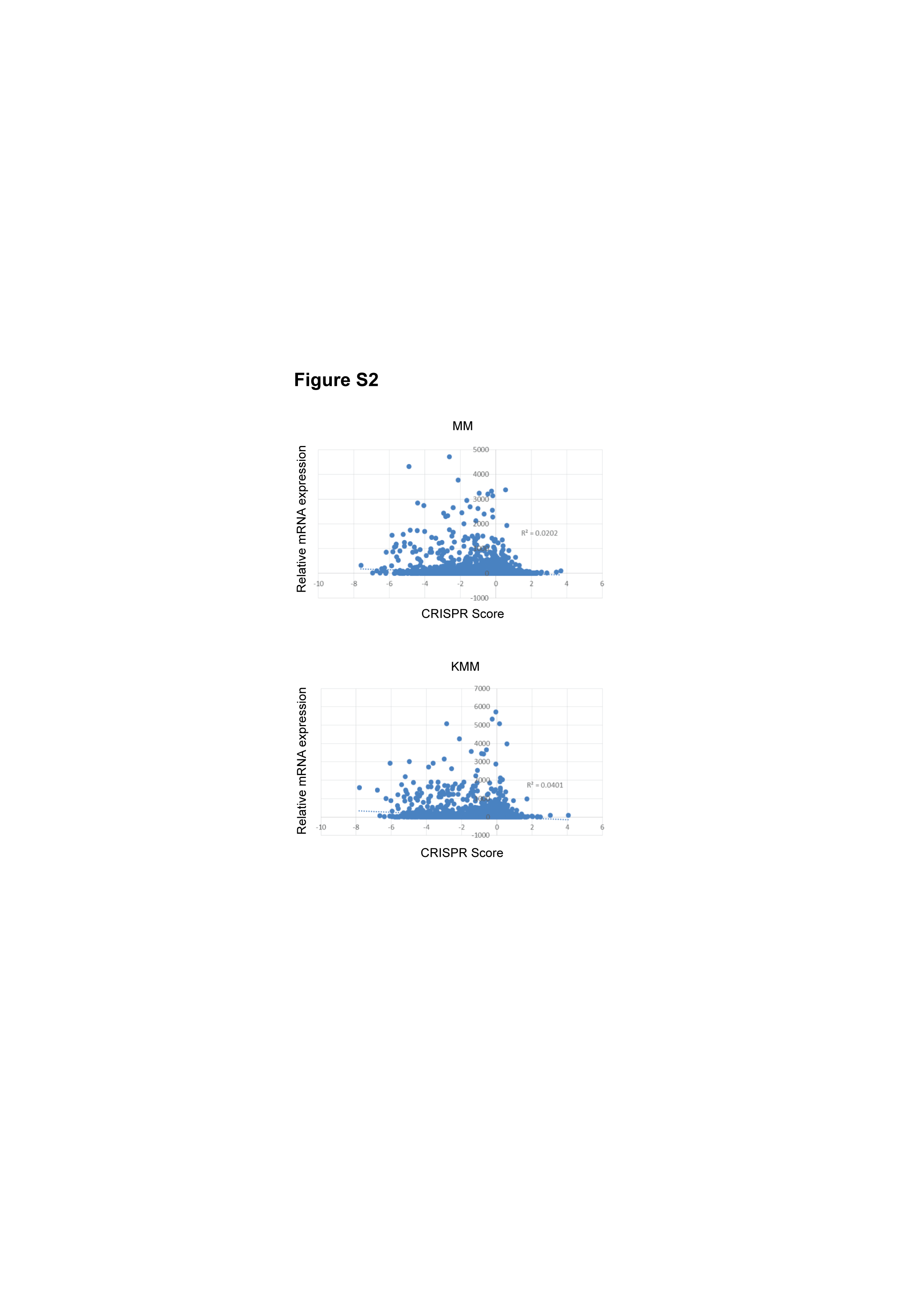

Supplement: FIG S2 [file mBio.00866-19-sf002.tif]

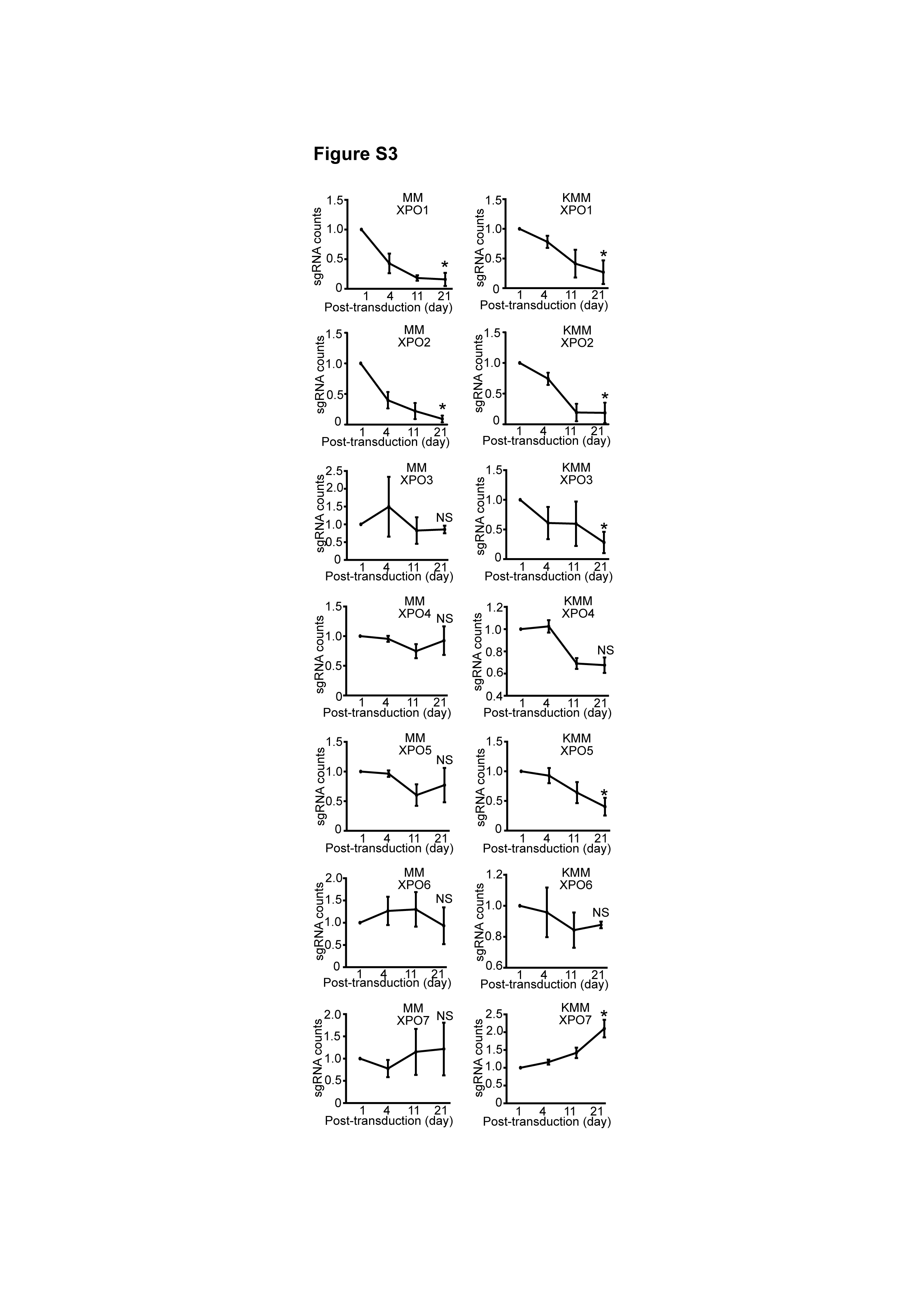

Supplement: FIG S3 [file mBio.00866-19-sf003.tif]
